# Cerebrotendinous Xanthomatosis: diversity of presentation and refining treatment with chenodeoxycholic acid

**DOI:** 10.1186/s40673-021-00128-2

**Published:** 2021-01-28

**Authors:** Mahjabin Islam, Nigel Hoggard, Marios Hadjivassiliou

**Affiliations:** 1 Academic department of Neurosciences, Sheffield Teaching Hospitals NHS Trust and University of Sheffield, Royal hallamshire Hospital, Glossop Road, Sheffield, UK; 2 Department of Infection, Immunity & Cardiovascular Disease, University of Sheffield / INSIGNEO, Sheffield, UK

**Keywords:** Cerebrotendinous xanthomatosis, Cholestanol, Chenodeoxycholic acid, Tendon Xanthomata, Early onset cataracts, CYP27A1, CTX, Ataxia

## Abstract

**Background:**

Cerebrotendinous xanthomatosis (CTX) is a rare but treatable neurometabolic disorder of lipid storage and bile acid synthesis. Whilst CTX is said to present with the classic triad of juvenile onset cataracts, tendon xanthomata and progressive ataxia, the diversity of presentation can be such that the diagnosis may be substantially delayed resulting in permanent neurological disability.

**Methods:**

A retrospective review of the clinical characteristics and imaging findings of 4 patients with CTX presenting to the Sheffield Ataxia Centre over a period of 25 years.

**Results:**

Although CTX-related symptoms were present from childhood, the median age at diagnosis was 39 years. Only 1 of the 4 cases had tendon xanthomata, only 2 cases had juvenile onset cataracts and 3 had progressive ataxia with one patient presenting with spastic paraparesis. Serum cholestanol was elevated in all 4 patients, proving to be a reliable diagnostic tool. In addition, cholestanol was raised in the CSF of 2 patients who underwent lumbar puncture. Despite treatment with chenodeoxycholic acid (CDCA) and normalization of serum cholestanol, CSF cholestanol remained high in one patient, necessitating increase in the dose of CDCA. Further adjustments to the dose of CDCA in the patient with raised CSF cholestanol resulted in slowing of progression. Two of the patients who have had the disease for the longest continued to progress, one subsequently dying from pneumonia.

**Conclusion:**

A high index of suspicion for CTX, even in the absence of the classical triad is essential in reaching such diagnosis. The earlier the diagnosis and treatment, the better the outcome.

## Introduction

Cerebrotendinous xanthomatosis (CTX) is a treatable neurometabolic disorder of lipid storage and bile acid synthesis. Mutations of the CYP27A1 gene result in deficiency of sterol 27-hydroxylase, an essential enzyme for conversion of cholesterol to chenodeoxycholic (CDCA) and cholic acids [1]. This results in reduced levels of Chenodeoxycholic acid; the process interrupts the feedback regulation of cholesterol 7-alpha-hydroxylase, which is the rate-limiting step in bile acid synthesis. The overall effect of this interruption is formation of cholestanol which is a metabolite of cholesterol that cannot be excreted. It therefore accumulates in plasma and is deposited in various lipophilic tissues such as brain with a preference for the cerebellum, eyes, resulting in cataracts and tendons resulting in tendon xanthomata [2].

Worldwide, more than 300 patients have been reported with a variety of mutations. An estimated prevalence of CTX in Caucasian population is 1:50,000 (patients with R362C CYP27A1 mutation), however, there is a strong possibility of under diagnosis due to its diverse manifestations [3]. Clinical presentation remains variable, even in patients within the same family or between identical twins [4]. CTX can present with any of the following symptoms: Infantile onset diarrhoea, childhood and juvenile onset cataracts, adolescent to young adult-onset tendon xanthomata, and childhood or adult-onset progressive neurologic dysfunction with one or more of the following: cerebellar ataxia, pyramidal and extrapyramidal signs, learning difficulties and intellectual disability, behavioural and psychiatric disturbances, dementia, peripheral neuropathy, and seizures [5]. The classic triad of this syndrome consists of early onset cataracts, tendon xanthomata, predominantly involving Achilles tendons, and cerebellar ataxia. We present here four cases of CTX with variable clinical presentations. We also discuss how to optimize treatment with chenodeoxycholic acid based on CSF measurements of cholestanol, something that has never been reported previously.

## Methods

This report is based on a retrospective observational case series of all patients with CTX regularly attending the Sheffield Ataxia Centre, Sheffield Teaching hospitals NHS Trust, Royal Hallamshire Hospital, Sheffield, UK. The South Yorkshire Research Ethics Committee has confirmed that no ethical approval is indicated given that all investigations/interventions were clinically indicated and did not form part of a research study. The Sheffield Ataxia Centre cares for over 2500 patients with progressive ataxia and these are the only 4 patients with CTX highlighting the rarity of the condition. In three patients the diagnosis of CTX was made at the Sheffield Ataxia Centre, based on clinical suspicion and subsequent cholestanol and genetic testing, whilst the 4th patient was diagnosed with CTX elsewhere and referred to the Sheffield Ataxia Centre for consideration of treatment. All patients received treatment with CDCA. Patients were followed up on a 6–12 monthly basis and serum cholestanol and MR Spectroscopy was used as a monitoring tool.

## Cases

### Patient 1

A 42-year-old man was referred to the Sheffield Ataxia Centre with a history of early onset cataracts, gait instability and cognitive difficulties. At the age of 8, his parents became concerned about his poor vision which may have contributed to his underperformance at school. He first came to medical attention because of tonic clonic seizures. An EEG revealed frequent general bursts of rhythmic 2–3 Hz activity but no lateralization. His performance at school deteriorated further. He suffered from severe anxiety and became very disruptive in the classroom resulting in social isolation. He was referred to a special school. At the age of 16, he was reviewed by a paediatric neurologist who found him to have pes cavus and bilateral increased tone in lower limbs, intention tremor in both arms and truncal ataxia. No diagnosis was made at that time. His visual disturbance was managed with visual aids until the age of 24 when he had bilateral extra capsular cataract extraction. His epilepsy was well controlled with phenytoin and he managed his day to day activities with support from his carer until the age of 35. There was no family history of any neurological problems.

**Fig. 1:**
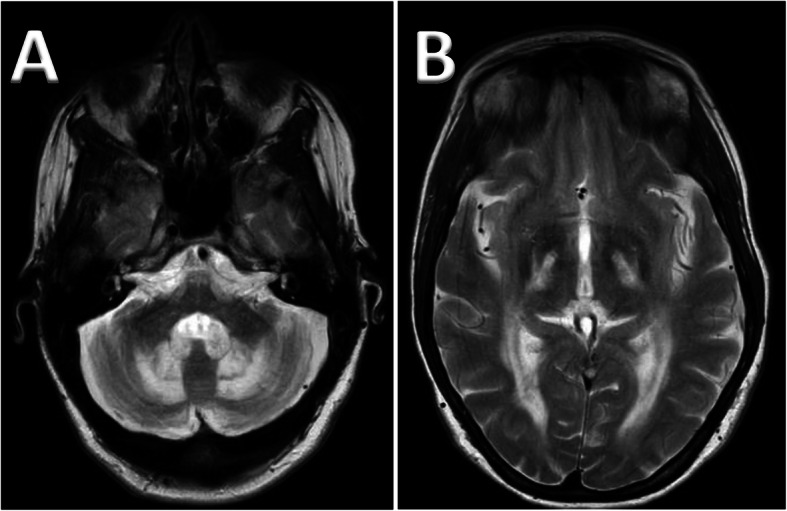
Axial T2 weighted imaging of patient 1, showing bilateral hyper intense lesions involving the dentate nuclei and the deep cerebellar white matter (**a**). Supratentorial imaging (Patient 2) shows white matter tract involvement, specifically of the corticospinal tract and optic radiation (**b**)

Examination revealed nasal speech, severe dysarthria, marked ataxia of limbs and gait and tendon xanthomata in both the Achilles tendons. His carer highlighted the deteriorating cognition resulting in poor memory, concentration and diminished intellectual abilities, all gradually worsening over many years. The suspicion of CTX was based on the typical clinical findings. Serum cholestanol was elevated at 53 μmol/L (normal range 3–16 μmol/L). At the time genetic testing for CTX was not readily available. For confirmation, examination of plasma and urinary bile acids were done to identify abnormal bile acid intermediates, notably glucuronides of pentols and hexols. His urinary bile acid analysis showed grossly abnormal peak of Cholestane-Pentol-Glucuronide, a characteristic abnormality seen in CTX. Other abnormalities included increased glucuronides of cholestane-tetrol, hexol and heptol as well as taurine and glycine conjugates of 23-hydroxycholate. MR imaging showed abnormal white matter primarily affecting the cerebellum (Fig. 1 a).

Despite the significant neurological disability, he was started on CDCA at a dose of 750 mg/day. His cholestanol level gradually reduced (53 μmol/L, 12 μmol/L, 7 μmol/L) over a period of 1 year. Clinically, there was a slight objective improvement in his speech, based on clinical examination and comments by his carers. He had a PEG tube inserted at the age of 44 due to recurrent aspirations and his communication had to be assisted with light writer. Although his biochemical parameters improved with CDCA, he continued to progress and died of pneumonia at the age of 45. Table 1 summarizes the clinical characteristics of all 4 patients.

**Table 1:** Summary of clinical characteristics in 4 patients with CTX

Patients	Sex	Age at symptom onset	Age at diagnosis	Infantile onset Diarrhoea	Intellectual Disability	Tendon Xanthomata	Early onset cataract
Patient 1	M	8	42	No	Yes	Yes	Yes
Patient 2	F	8	37	Yes	Yes	No	Yes
Patient 3	M	15	37	No	Yes	No	No
Patient 4	M	25	41	Yes	No	No	No
**Patients**	**Pyramidal signs**		**Extrapyramidal signs**	**Epilepsy**	**Dementia**	**Neuropathy**	**Osteoporosis**
Patient 1	Yes		No	Yes	Yes	Yes	No
Patient 2	Yes		No	No	No	No	Yes
Patient 3	Yes		Yes	No	No	Yes	No
Patient 4	Yes		No	No	No	Yes	No

### Patient 2

A 37-year-old lady presented primarily because of deteriorating balance and a background of mild cognitive problems. She was labelled as having Asperger’s syndrome. Notable in her past medical history was early onset cataracts at the age of 8 years which were extracted when she was 9. She suffered from diarrhoea when she was a child.

Her mother reported that she had been clumsy from a young age. She reached normal developmental milestones, particularly in regards to motor development. Apart from early onset hypertension and osteoporosis she was reasonably well and mobile until the age of 34 when she became unsteady on her feet and extremely fatigued. She started having frequent falls and fractured her left ankle following a fall. She had 3 siblings none of whom had any neurological symptom. Both parents were asymptomatic.

Neurological examination revealed limb and gait ataxia, very brisk reflexes and extensor plantars but no tendon xanthomata. She was unable to do tandem walk.

MR imaging showed extensive white matter changes in cerebellum, high signal in inferior olives bilaterally, there was also increased signal changes supratentorially, involving corpus callosum, internal capsule and corona radiata, and white matter tract specifically of the corticospinal tract and optic radiation (Fig. 1b).

Because of the history of early onset cataracts, diarrhoea and ataxia, serum cholestanol was measured and found to be high at 145 μmol/L. A diagnosis of CTX was made and she was started on CDCA 750 mg per day. She initially improved; physiotherapy assessments showed improvement in 10 m walk test and single leg stand test. Serum cholestanol normalised with the treatment but this took 2 years.

Despite having normal serum cholestanol, her mobility then started to deteriorate. She became increasingly more dysarthric and developed swallowing difficulties. Analysis for 7 alpha hydroxy 4-cholesten-3-one in plasma (another way of measuring the effectiveness of CDCA) showed it to be within normal limits (46 nmol/L, normal level < 100 nmol/L). The advice based on this test was that she should continue with the current dose of CDCA.

Six years after the start of treatment with CDCA at a dose of 750 mg/day, CSF cholestanol was measured and was found to be five times higher when compared to CSF of healthy controls. As a result, the dose to CDCA was increased to 1000 mg per day. Repeat CSF after a year on the higher dose, showed the CSF cholestanol to have reduced significantly but still higher than normal controls. Unfortunately the patient could not tolerate a higher dose of CDCA, and compliance became an issue due to nausea and vomiting. The family were reluctant for her to have any further medical interventions (e.g. repeat CSF studies and imaging) and currently she is cared for at home by her parents. Her condition has deteriorated further but the rate of deterioration was felt to be slower (according to her family who cared for her on a daily basis) after increasing the dose of CDCA (Table 2).

**Table 2:** Summary of biochemical and genetic investigations

	serum cholestanol at diagnosis normal < 5 μmol/L	CSF cholestanol reference range (0.252–0.376 μmol/L) based on healthy controls)	serum Cholestanol after treatment	Urine Bile acid alcohol	Genetic Confirmation
Patient 1	53	not done	7	High	Not done
Patient 2	145	1.06 μmol/L on standard dose, 0.49 μmol/L after CDCA dose increase	10	High	CYP27A1: c.1184 + 1G > A(;) 1263 + 1G > A, p.(?)(;)(?)
Patient 3	125	2.5 (μmol/L) before treatment	41	Not done	CYP27A1c.157del,p.(Arg53fs)
Patient 4	112	not done	not repeated as yet	Not done	CYP27A1: c.[1183 > T];[1183C > T], p[(Arg395Cys)];p[(Arg395Cys)]

### Patient 3

A 37-year-old gentleman was referred to the Sheffield Ataxia Centre with worsening speech and increased tendency to fall. His symptoms started at the age of 15. He denied any visual problem. He developed urgency and frequency of micturition. By the time he was seen, he was largely confined to a wheelchair and requiring help from at least one person to be able to walk. He had a background of learning difficulties, attending a special school from a young age. He denied any diarrhoea or other bowel symptom. He came from a family of three brothers and two sisters none of whom had similar symptoms. Both parents, in consanguineous marriage, were in their eighties and well.

Neurological examination showed no evidence of cataract or tendon xanthomata. His speech was very dysarthric, and he had limb and gait ataxia, increased tone, pathologically brisk reflexes in both legs with bilateral extensor plantar responses. He also had tremor affecting both arms.

**Fig. 2:**
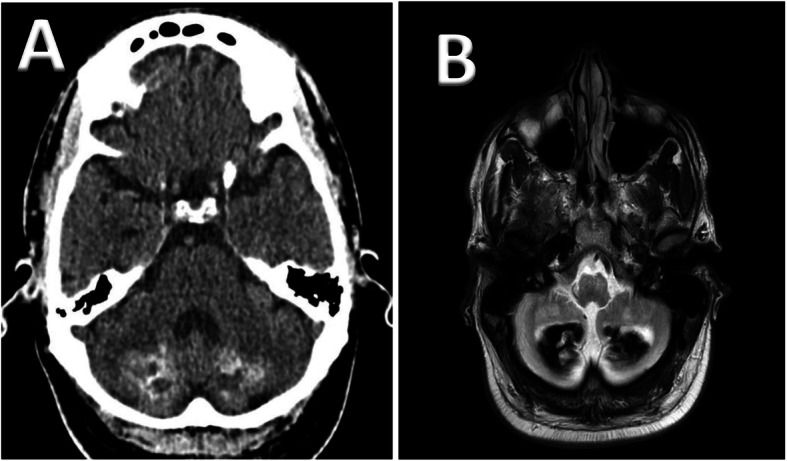
CT (**a**) and MRI (**b**) images from Patient 3 showing evidence of cerebellar calcification which although rare can be a feature of CTX

MRI of the brain showed signal change within the cerebellar hemispheres, which was associated with significant volume loss. There was a combination of low signal change suspected to be due to calcification (confirmed on CT – Fig. 2a) and raised signal change in the rest of the cerebellar hemispheres extending into the cerebellar peduncles bilaterally (Fig. 2b).

A diagnosis of CTX was not immediately suspected based on the clinical and imaging findings but the early onset of ataxia with parental consanguinity was strongly suggestive of an autosomal recessive genetic cause. Genetic testing using next-generation sequencing (NGS) showed him to be homozygous for the CYP27A1c.157del,p.(Arg53fs) mutation, confirming the diagnosis of CTX.

At the time of diagnosis, serum cholestanol was high (125 μmol/L) as expected. Baseline CSF cholestanol sample (before treatment) revealed high titre of 2.5 μmol/L when compared to healthy control CSF samples (0.252–0.376 μmol/L). He was started on CDCA at a dose of 750 mg/day. After 6 months of treatment, he and the family reported improvement in the severity of the tremor and this was confirmed on neurological examination; however, gait instability remained unchanged although his serum Cholestanol level dropped to 41 μmol/L. No further updates are yet available.

### Patient 4

This 27-year-old man presented with a 3-year history of stiffness and then weakness and sensory disturbance affecting both legs. He had a history of infantile onset diarrhoea which persisted throughout his life. He was extensively investigated for coeliac disease, but biopsy proved to be negative. He had normal developmental milestones and had been quite active in sports during childhood and in his early 20’s. In fact he was playing Taekwondo until the onset of his symptoms.

Examination revealed nystagmus on lateral gaze, brisk jaw jerk, increased tone bilaterally in arms and legs with pathologically brisk reflexes, and extensor plantar responses. He walked with a rather spastic gait and had bilateral pes cavus. His parents were in good health, with the only medical problems being coeliac disease of the mother. He was thought to have hereditary spastic paraparesis (HSP). Initial limited genetic testing for HSP was negative. He was followed up in neurology and managed with antispasmodics. His condition gradually deteriorated and he eventually ended up wheelchair bound due to the severity of the spasticity.

The stored DNA sample was again tested using extended HSP panel. He was found to be homozygous for a pathogenic mutation of the CYP27A1 gene. His cholestanol level was 112 μmol/L at baseline.

**Fig. 3:**
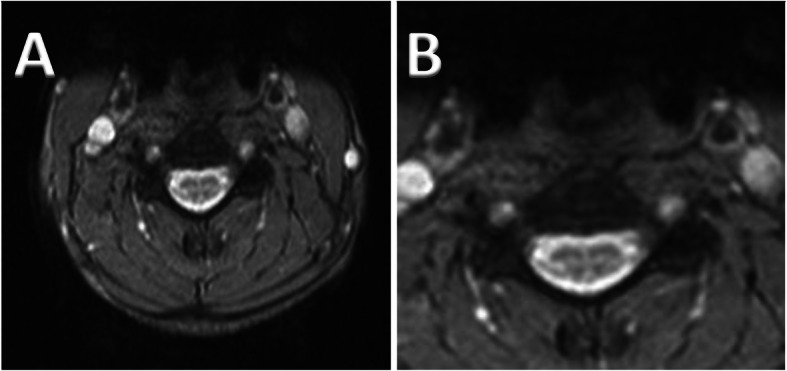
Axial T2 MRI spinal images (Patient 4) showing signal changes affecting mainly lateral Corticospinal tracts (magnified in image **b**)

MRI of brain showed cerebellar atrophy, significantly worse in the hemispheres than the vermis with signal change around the dentate nucleus extending into the cerebellar peduncles. His spinal MRI also showed signal changes mainly involving lateral corticospinal tracts (Fig. 3a, b).

He has been started on chenodeoxycholic acid recently and is under review.

## Discussion

CTX is an autosomal recessive lipid storage disorder caused by mutations in the CYP27A1 gene which leads to abnormal deposition of cholestanol in different lipophilic tissues resulting in various neurological and non-neurological manifestations. It was first described in 1937 by Van Bogaert and colleagues [6]. Chenodeoxycholic acid replacement, the treatment of choice, was reported first in 1975 by Salen et al. and subsequently by Berginer [7, 8].

We describe here a series of 4 patients with CTX who presented with diverse manifestations but eventually were diagnosed with this rare condition. In addition to the clinical characteristics, we provide detailed imaging data and our experience in the treatment with CDCA aided by CSF monitoring of cholestanol. This variability in presentation has been considered to be the cause of delay in diagnosis. Whilst in the presence of the classic triad of early onset cataracts, tendon xanthomata and progressive ataxia often with pyramidal signs all neurologists should be alerted to the possibility of CTX, our cohort shows that this triad was only seen in 25% of cases. This diagnostic triad fails to highlight another important feature of this disease which is the cognitive deficits that seem to be prevalent at a young age interfering with schooling and being misdiagnosed as behavioral or psychological problems or, as in one case here Asperger’s syndrome. It would be advisable to test (using serum cholestanol) all patients with early onset cataracts even in the absence of any neurological deficits to facilitate earlier diagnosis. The same is true for patients with clear evidence of tendon xanthomata. Such an approach may facilitate early diagnosis and treatment and may prevent permanent neurological disability as was the case in all 4 of our patients [5]. The mean age at diagnosis of CTX in this cohort was 39 years whilst the mean age at symptom onset was 14. This means that the mean delay in the diagnosis was 25 years.

As described by many, the importance of diagnosing this neurodegenerative condition is because it is potentially treatable. The treatment can reverse, stabilize, or prevent accumulation of cholestanol in CNS slowing the development or stopping the progression of neurological symptoms [5, 9]. A cross-sectional observational study demonstrated worse outcome and significant limitation in ambulation and cognition in patients with CTX diagnosed after the age of 25 despite treatment with chenodeoxycholic acid [10].

To aid early diagnosis, Mignarri et al. devised a suspicion index composed of weighted scores assigned to different indicators which follows a diagnostic flow chart to aid early detection [11]. In this scoring system, very strong indicators include family history (sibling with CTX) and tendon xanthomata. Other parameters include consanguineous parents, juvenile cataracts, childhood-onset chronic diarrhoea, prolonged unexplained neonatal jaundice or cholestasis, ataxia and/or spastic paraparesis, dentate nuclei signal alterations on MRI, intellectual disability and/or psychiatric disturbances. Moderate criteria include early osteoporosis, epilepsy, parkinsonism and polyneuropathy. All 4 cases described here, scored 100 or more using the suspicion index tool developed by Mignarri et al. and qualified for serum cholestanol measurement. This supports the use of this tool for early diagnosis.

CDCA has been shown to be very effective in reducing the serum cholestanol in CTX patients and this has been our experience with this cohort [12]. Yet 2 of our patients continued to progress after some initial minor improvement. One patient died due to pneumonia at the age of 45. He was extremely disabled, confined to a wheelchair and required PEG feeding. In patient 2, progressive clinical deterioration and lack of improvement despite normalisation of serum cholestanol let us to examine the CSF. We were able to demonstrate that the CSF cholestanol remained high despite normal serum cholestanol and that increasing the dose of CDCA reduced CSF cholestanol further.

Previous work suggests that the level of CSF cholestanol can be as high as 20 times the normal healthy population and that treatment with CDCA reduces CSF cholestanol by three fold [13]. The question here, is why does normalisation of serum cholestanol not accompanied by normalisation of CSF cholestanol? Could this be the reason why some patients do not respond that well to CDCA? We were able to show that adjustments to the dose of CDCA can result in further decrease of the CSF cholestanol. The clinical benefit was minimal probably because the disability was so severe.

The precise pathophysiology of neurological damage in CTX remains unclear. Some postulate that raised level of apolipoprotein B concentration in CSF permits increased transportation of cholesterol and cholestanol across the blood-brain barrier. Accumulation of cholestanol at a high concentration in the brain tissue initiates apoptotic pathways which eventually lead to neuronal death. Chenodeoxycholic acid treatment re-establishes selective permeability of the defective blood brain barrier and normalizes the level of sterols and apolipoprotein in CSF, therefore minimizes further damage [13]. However, the existing deposits of cholestanol may still perpetuate the apoptosis. Of interest, is the observation that cholestanol deposition seems to have a predilection for the cerebellum, at least in those classic cases*.* It remains obscure why this should be the case or why in some cases (less common) the deposition affects primarily the spinal cord. This predilection of cholestanol deposition in the cerebellum was suggested to be the result of low abundance of CYP46A1, the principal cholesterol 24-hydroxylase, in the cerebellum [14].

A retrospective study of 56 patients with CTX by Bianca et al. concluded that patients who are diagnosed and treated before the age of 24 had complete resolution of their neurological symptoms and prevented the development of new neurological symptoms at followed up [5]. This emphasises the importance of early diagnosis and treatment in preventing long term disability.

The increased availability of genetic testing and the inclusion of CTX in both ataxia and HSP panels is likely to aid the diagnosis of CTX. As noted in one of our cases, spastic paraparesis can be the first presentation of CTX, therefore should be considered as a part of the differential diagnosis of chronic myelopathy particularly if there is signal change in the cord on spinal imaging [15]. However, there is a need for a high index of suspicion in younger population with a much more subtle phenotype that may just include learning and cognitive difficulties often with behavioural problems for which cholestanol estimation should be part of the diagnostic workup.

## Data Availability

Not Applicable.

## Abbreviations

CTX: Cerebrotendinous xanthomatosis
CDCA: Chenodeoxycholic acid
MRI: Magnetic resonance imaging
CT: Computed Tomography
NGS: Next-generation sequencing
EEG: Electroencephalogram
PEG: Percutaneous endoscopic gastrostomy
HSP: Hereditary Spastic Paraparesis
